# Joint Simon effect in movement trajectories

**DOI:** 10.1371/journal.pone.0261735

**Published:** 2021-12-29

**Authors:** Ekaterina Sangati, Marc Slors, Barbara C. N. Müller, Iris van Rooij

**Affiliations:** 1 Faculty of Philosophy, Theology and Religious Studies, Radboud University Nijmegen, Nijmegen, The Netherlands; 2 Behavioural Science Institute, Radboud University Nijmegen, Nijmegen, The Netherlands; 3 Donders Institute for Brain, Cognition, and Behaviour Centre for Cognition, Radboud University Nijmegen, Nijmegen, The Netherlands; University College London, UNITED KINGDOM

## Abstract

In joint action literature it is often assumed that acting together is driven by pervasive and automatic process of co-representation, that is, representing the co-actor’s part of the task in addition to one’s own. Much of this research employs joint stimulus-response compatibility tasks varying the stimuli employed or the physical and social relations between participants. In this study we test the robustness of co-representation effects by focusing instead on variation in response modality. Specifically, we implement a mouse-tracking version of a Joint Simon Task in which participants respond by producing continuous movements with a computer mouse rather than pushing discrete buttons. We have three key findings. First, in a replication of an earlier study we show that in a classical individual Simon Task movement trajectories show greater curvature on incongruent trials, paralleling longer response times. Second, this effect largely disappears in a Go-NoGo Simon Task, in which participants respond to only one of the cues and refrain from responding to the other. Third, contrary to previous studies that use button pressing responses, we observe no overall effect in the joint variants of the task. However, we also detect a notable diversity in movement strategies adopted by the participants, with some participants showing the effect on the individual level. Our study casts doubt on the pervasiveness of co-representation, highlights the usefulness of mouse-tracking methodology and emphasizes the need for looking at individual variation in task performance.

## 1 Introduction

Research on social cognition has advanced by moving beyond scenarios of passive observation of other’s actions or instructed imitation tasks to situations of joint action. Based on one of the most popular tasks in this research, the so-called Joint Simon Task (JST), it has been argued that humans co-represent each other’s tasks during joint action [1]. This proposal, which we will refer to as the *social account*, has not gone unchallenged. Some cognitive scientists have proposed that the effects observed in the JST need not be seen as evidence of a specifically social cognitive mechanism of ‘co-representation’, but instead can be explained by an appeal to domain-general cognitive processes (the *domain-general account*; [2]). So far, this debate has been limited to trying to come up with competing mechanisms that could underlie the effect obtained in variations of the same basic experimental paradigm. A question that has not been addressed, however, is if this phenomenon, if it indeed occurs, is as pervasive and automatic as has been assumed in the social account. In this paper we set out to contribute to the running debate by targeting this latter question.

**Fig 1:**
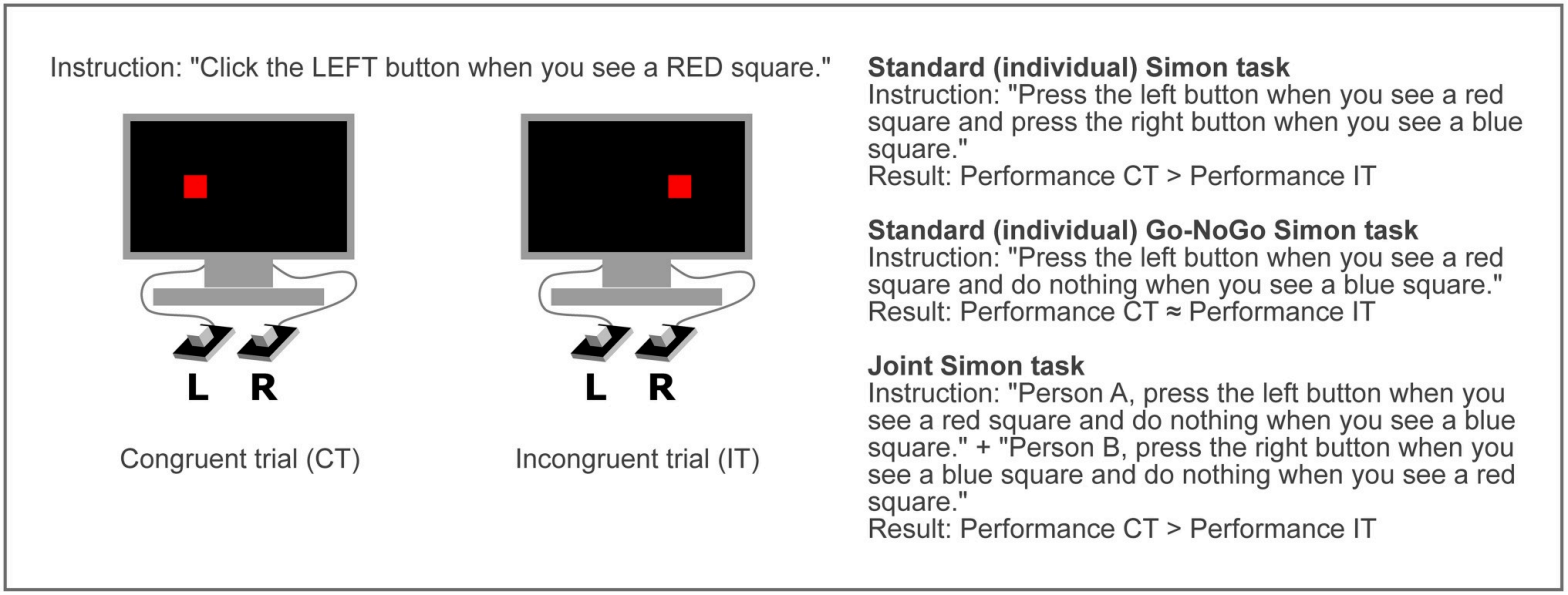
Button-based Simon task setup. On the left two trials are shown: a congruent trial in which the red cue is located on the same side as the required response and an incongruent trial in which the cue is located on the opposite side. On the right 3 conditions described in the main text are presented: SST, its Go-NoGo version and JST with their example instructions and typical findings.

The JST is an extension of the standard Simon Task (see illustration in Fig 1). In a standard, non-joint Simon task (SST) participants carry out spatially defined responses (e.g., push a left or right button) to stimuli defined by a task-relevant non-spatial dimension (e.g., shape or color) while ignoring their spatial dimension (e.g. stimuli themselves appearing on the left or right side of the screen). What is typically found in such an experiment is that incongruent trials (pushing the left button in response to the stimulus appearing on the right) lead to worse performance, that is, an increased number of errors and slower reaction time compared to congruent trials (pushing the left button in response to the stimulus on the left). This is commonly explained as a result of conflict in the response selection stage produced by an automatic encoding of stimulus spatiality which interacts with an active representation of two response alternatives. Accordingly, it has been found [3] that the Simon effect disappears if the participant is asked to perform a Go-NoGo version of the task in which they have to respond to only one type of stimulus with one type of a response. Arguably, this is due to the fact that if only one kind of response is required, it can be represented in a non-spatial way, for example as needing to push *a button*, not a *left* or *right button* and therefore no conflict with the spatiality of the stimulus can arise.

The JST has been designed by Sebanz and colleagues [1] to investigate how one’s own action planning is affected by the intentions, actions or tasks of another person. They reasoned that if two people are assigned complementary halves of the task and they are not affected by each other’s roles, there will be no Simon effect, just as in the individual Go-NoGo version of the task. However, the experiment showed that the Simon effect does occur in such a joint version, leading Sebanz and colleagues to conclude that “the action at the other’s disposal was represented and subject to automatic response activation by the irrelevant stimulus dimension” (p. 15), just as in the individual Simon task. Interestingly, what also emerged from the experiment is that blocking any auditory or visual information about the other’s responses did not alter the effect, suggesting that representing the other’s part of the task is a relatively high-level, “offline” process, i.e. it can be activated by mere knowledge of the task and the co-actor’s contribution to it, and unaffected by whether or not one witnesses the other’s actions (see also [4, 5] for corroborating but [6] for contrasting evidence).

The mechanism proposed to underlie the joint Simon effect (JSE) has since been dubbed *co-representation* and it became an ingredient of a broader social account of joint action, that also includes processes of action simulation and prediction, joint attention, perspective-taking, and mind-reading [7–9]. According to this account, solo and joint actions are functionally equivalent in that they rely on representations of task constraints and required actions, except that in the joint case, the task is represented as distributed among the participants, allowing one’s own actions to be coordinated with that of the co-actor. The resulting account is ‘social’ because it relies on specifically *social* representations whose content is something about the internal state of another intentional agent (her goals, intentions and actions specified by the task) and *social* mechanisms operating only in social contexts and influenced by social factors. Furthermore, it has also been suggested that the formation of co-representations is pervasive across situations and automatic, that is, “even if that leads to a decline in one’s own performance … people cannot help representing what other people do” [10, p. 101].

Of the three features of co-representation, i.e. its social, offline, and automatic nature, the most heated debate centered on the first one. The proponents of the social nature of co-representation have provided evidence that the JSE is more likely when the co-actor is an intentional agent [11–14] and that it is affected by a host of social factors [15–18]. However, a different group of researchers have demonstrated that JSE can be found with passive and even non-human co-actors [2, 19, 20] and that the co-actor does not seem to share all aspects of the task (e.g., the proportion of congruent and incongruent trials; [21]). The former casts doubt on the social nature of co-representation while the latter puts a limit on the extent of the overlap with the co-actor. In order to explain these results, a competing domain-general theory has been proposed, according to which the JSE is due to the presence of salient events that induce spatial coding of the perceived task environment and required actions [22]. A more general idea is that people navigate social situations not due to some specifically social mechanisms but by simply perceiving task environment differently than when they are on their own and therefore, the notion of task-sharing should be replaced by a more neutral term like task-shaping of the participants by an enriched context [23].

While the debate on the mechanisms underlying the JSE and joint action more broadly is undoubtedly important, we believe it risks losing sight of the big picture that inspires this research in the first place. What research on joint action ideally should explain is how real people in a variety of real-life situations manage to act together employing a range of possible strategies. If an account of joint action postulates a mechanism to explain an observed phenomenon, we should make sure that the phenomenon actually occurs in real-world interactions and it is not limited to, or worse, an artifact of an experimental paradigm. In other words, we should ask ourselves the question to what extent the phenomenon has ecological relevance.

Such reflection is especially important in light of recent evidence that JSE itself might not be as robust as previously thought. A meta-analysis conducted on 39 distinct studies that employed JST [24] found that (1) there is reason to believe there is a publication bias skewed towards studies that found a JSE, (2) restricting the analysis to studies with large samples removes the bias but reduces the overall effect size to *d* = 0.17. These two findings together indicate that the effect may not be reliably present and even when it is, its size is small. Karlinsky and colleagues caution that this may indicate a “limited ‘practical’ significance of this effect”. Here we would argue that it may also limit its “theoretical” significance: the unreliability of the JSE suggests that—leaving statistical bias aside—the evidence for the pervasiveness and automaticity of co-representation is weak at best.

In this study, we add to the discussion of pervasiveness of the JSE by taking a preliminary step towards exploring whether it is robust under variation of the experimental paradigm that incorporates some of the aspects of more naturalistic settings of embodied joint action. Specifically, we developed a paradigm in which participants can move continuously and have more real-time (“online”) information about the co-actor’s movements. This is motivated by the fact that in real life, joint action is typically done by people present in the same location and able to observe each other’s movements. Given the assumption that the JSE is driven by offline, automatic processes of co-representation, the JSE should remain robust under such variants. If it does not, then this would cast more doubt on the ecological relevance of the effect for embodied joint action (and the mechanisms postulated to explain it). Note that this moves in an opposite direction compared to previous research that attempted to answer whether JSE is affected by the presence of the co-actor. There a typical comparison was between a situation of people executing button presses while seated next to each other and the situations of *decreasing* access to information about the co-actor [4–6, 18, 25–27].

To emphasize this in other words: our study does not aim at pitching different accounts of mechanisms that underlie JSE, i.e., co-representation versus domain-general accounts, against each other. Instead, we probe the ecological plausibility of the co-representation account by modifying an existing prominent paradigm in a slightly more naturalistic version to test if indeed this postulated mechanism is as pervasive as seems to be implied by its presumed automaticity.

In our experimental paradigm, we employ a dynamic mouse-tracking methodology [28, 29]. In this methodology participant is carrying out a choice task by selecting responses via moving a computer mouse cursor to one of the specified locations on the screen. The mouse movement is recorded at high sampling frequency and the resulting trajectories analyzed in various ways. This allows for a more fine-grained insight into the evolution of decision over time than, for instance, simply measuring reaction times. What is typically found in studies that employ mouse-tracking is that in certain conditions trajectories reveal a deviation toward an unselected response, indicating its covert activation. This approach has been used in a variety of settings, such as social perception [30], language processing [31], Implicit Association Test measure [32] and even theory of mind [33].

**Fig 2:**
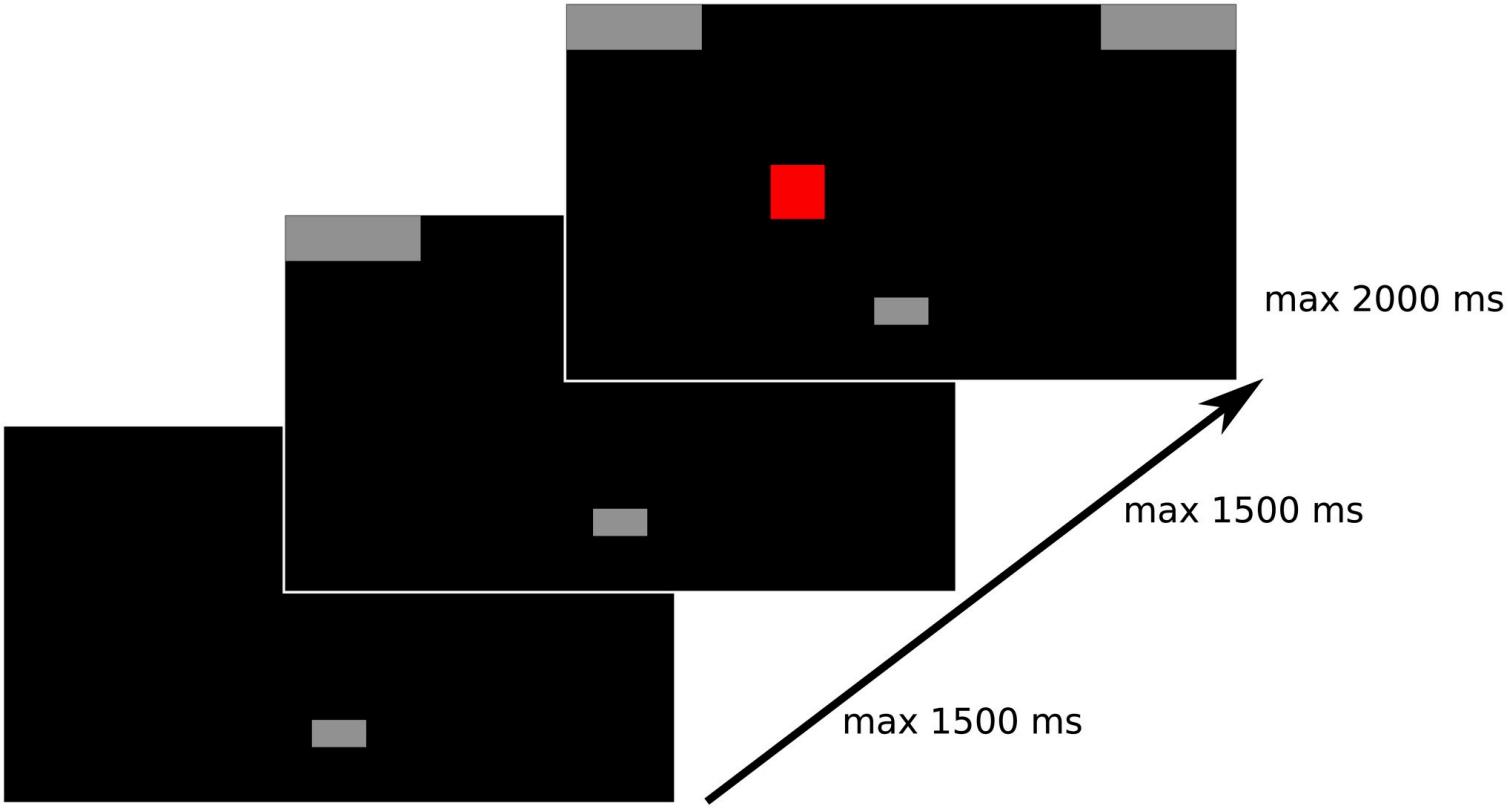
Trial time course. The button in the bottom center of the screen is the “start button”. The upper corners are response areas. The stimuli occur in the center portion of the screen after variable delay. Each stage of the trial has a deadline, missing which leads to an abortion of a given trial and a beginning of a new trial.

Adapting the mouse-tracking methodology to the Simon task means that instead of clicking right or left buttons in response to the Simon task stimuli, participants execute responses by moving the cursor from a starting position to one of the response boxes presented on a screen (see Fig 2). Previously it has been demonstrated by [34] that by using such a trajectory-based response modality the standard (individual) Simon effect can be replicated. Their results showed that in addition to slower RT in incongruent trials, the mouse trajectory is also affected by exhibiting greater curvature towards the wrong response box, indicating an implicit attraction towards the unselected competing response, as explained above [29]. If this effect were to be replicated in a JST version of the mouse-tracking paradigm it would be the continuous (mouse-tracking) analog of the classical (key press) JSE.

To our knowledge there has been only one study that was partially implemented to investigate mouse trajectories in a JST [35]. However, in this study both participants performed a full version of the standard Simon task, not a complementary Go-NoGo task, which prevents any interpretation as to whether the effects observed are due to the individual spatial compatibility effect or due to anything that has to do with joint action. Additionally, the study setup involved participants responding in two different modalities, one executing responses with the mouse, while their co-actor was still pressing buttons. First, it is unclear how such a setup could be integrated into one potential co-representation. Second, it allows one to investigate only the effect of executing continuous actions, not observing the actions of the co-actor.

In an attempt to improve the interpretability of the joint mouse-tracking task, we made the following modifications, implementing four different mouse-tracking variants of the (Joint) Simon task in three successive experiments with different participants.

Our Experiment 1 was designed to test if we could replicate the mouse-tracking Simon effect observed by [34]. Experiment 2 tested whether in an individual Go-NoGo mouse-tracking task, in which each participant responded to only one cue, the effect is indeed absent as has been observed in the key press version of the task [1]. Experiment 3 was the main target of our study. It employed a traditional JST design, performed by pairs of participants, in which one participant responded to one cue and the other to the complementary cue. In our study both participants performed a mouse-tracking implementation of the task. We tested two versions of this task: an online version in which participants had online information about the others’ actions: each participant saw both their own and their partner’s cursor on the screen; and an offline version in which participants only saw their own cursor. With Experiment 3 we wanted to test whether or not a mouse-tracking JSE occurs under these conditions.

If we find a mouse-tracking JSE, it would increase the likelihood that the effect is robust and generalizes to a paradigm with more continuous response format, which seems a minimal condition for it to apply also in life-like situations of joint action. Furthermore, it would give researchers an additional tool to investigate the mechanisms underlying the JSE, such as the ability to observe an unfolding of action over time, including a decision to inhibit the response. If, however, we find no effect in our Experiment 3, this puts into question the robustness and generalizability of the effect. Such a result would bear on the nature of the mechanisms that are postulated to underlie the effect, specifically the presumed pervasive, automatic, social and offline nature of co-representation.

All measures, manipulations, and exclusions in the study are disclosed. Implementations of the experiment and data analysis reported in this paper are made publicly available. Experiment code: https://gitlab.com/lumina.noctis/jse_experiment. Analysis code: https://gitlab.com/lumina.noctis/jse_data_analysis.

## 2 Experiment 1

The aim of the first experiment was to replicate a previous study by Scherbaum and colleagues [34] in order to ensure that the recreated setup delivers comparable results for the individual Simon condition. We have followed the description of their experimental conditions with the exception of employing different type of stimuli: colored squares instead of white arrows. As such, our Experiment 1 serves as a generalization test for the individual Simon effect in mouse trajectories. We predicted that participants in incongruent trials will exhibit longer reaction times and movement trajectories curved towards the incorrect response.

### 2.1 Methods

#### 2.1.1 Participants

Twenty participants from the Radboud University participants pool (14 women; ages between 19 and 30 years, mean age 23.9) were recruited for the first experiment. The number of participants was based on the previous study by Scherbaum and colleagues [34] (cf. [36]) and is justified by a large number of trials that each participant performs (640). A sensitivity power analysis for our main paired one-tailed t-test indicated that the minimum effect size we could detect was a medium effect of *d* = .58, assuming an *α* of 0.05 and power of 0.8. All participants were right-handed, had normal or corrected-to-normal vision and were not aware of the purpose of the experiment. They were paid 10 euros for their participation. The study was approved by the institution’s local ethics committee (ECSW2015–1105-309) and written informed consent was obtained from each participant.

#### 2.1.2 Apparatus and stimuli

All participants were seated about 60 cm in front of a 24 inch computer screen (a resolution of 1920x1080 pixels, 120 Hz refresh frequency) and performed the task on their own. We used Psychophysics Toolbox 3 [37] as presentation software running in Matlab 2016a on Windows 7. Target stimuli were red and blue square boxes presented in turn on a black background. They had a size of 3.82° and an eccentricity of 7.16° at 60 cm distance. The top left and right of the screen contained gray response boxes of width 9.55°. There was a small gray rectangle (3.82 width) displayed at the bottom center of the screen that played the role of a “start button”. Responses were executed by moving a standard computer mouse (Logitech G500S). Mouse positions were sampled with a frequency of 92 Hz and recorded in each trial from the presentation of the start rectangle until response.

#### 2.1.3 Task and procedure

Participants were asked to respond to the color of a presented square by clicking on the assigned response box. The association between the color of the square (blue or red) and the response box (left or right) was counter-balanced across participants.

Each trial consisted of three stages (see Fig 2). In the first stage participants were asked to click on the small gray box at the bottom of the screen in order to start the trial within a deadline of 1.5s. After the start click, two gray response boxes appeared on the screen and participants had to start moving upward within a deadline of 1.5s. If they started moving as requested and crossed a specific y-threshold (unknown to the participants), a color square appeared on the left or right side of the screen. Participants were asked to click on the response box corresponding to the color of the cue and irrespective of the cue’s location within a deadline of 2s. If participants missed any of the deadlines, the trial ended, the screen turned black briefly and a new trial began.

Each experimental run started with a presentation of instructions on the screen and a practice block of 40 trials. The first 10 practice trials involved no deadlines and ended with feedback as to the time and correctness of the response. The next 10 practice trials involved deadlines and feedback. The last 20 practice trials involved deadlines and no feedback. The experiment consisted of 2 blocks and 320 test trials per block. The color and location of the cue was balanced within each block by pseudo-randomization. A complete procedure with debriefing took 45 minutes.

#### 2.1.4 Data preprocessing

Following recommendations by [38] we pre-processed the mouse trajectories by aligning them for common starting position (horizontal middle position of the screen, 540 pixels), flipping them to the same direction and normalizing to 100 equal time slices. We used a combination of self-written code as well as a specific mouse trajectory analysis package [39] for data analysis. The raw collected data is archived at https://doi.org/10.17026/dans-xgy-wzvt.

### 2.2 Results

Trials were coded as Congruent when the cue appeared on the same side of the screen as the response box to which the cue color was assigned and Incongruent when the cue appeared on the opposite side. One participant was found to have misunderstood the instructions (they responded correctly on only 2% of the trials) and her/his data was removed from further analysis. Of the remaining data, trials were removed if any of the deadlines was missed, an incorrect response was given, a sampling interval (the time between recording of the coordinates) was ≤91 Hz or ≥93 Hz or a total reaction time was 3*MAD* above the sample median value. Altogether this led to the elimination of around 5% of the remaining data (i.e., not counting the fully removed participant). See Table 1 in S1 File for the explanation of how data was removed in all the experiments.

#### 2.2.1 Reaction times

**Fig 3:**
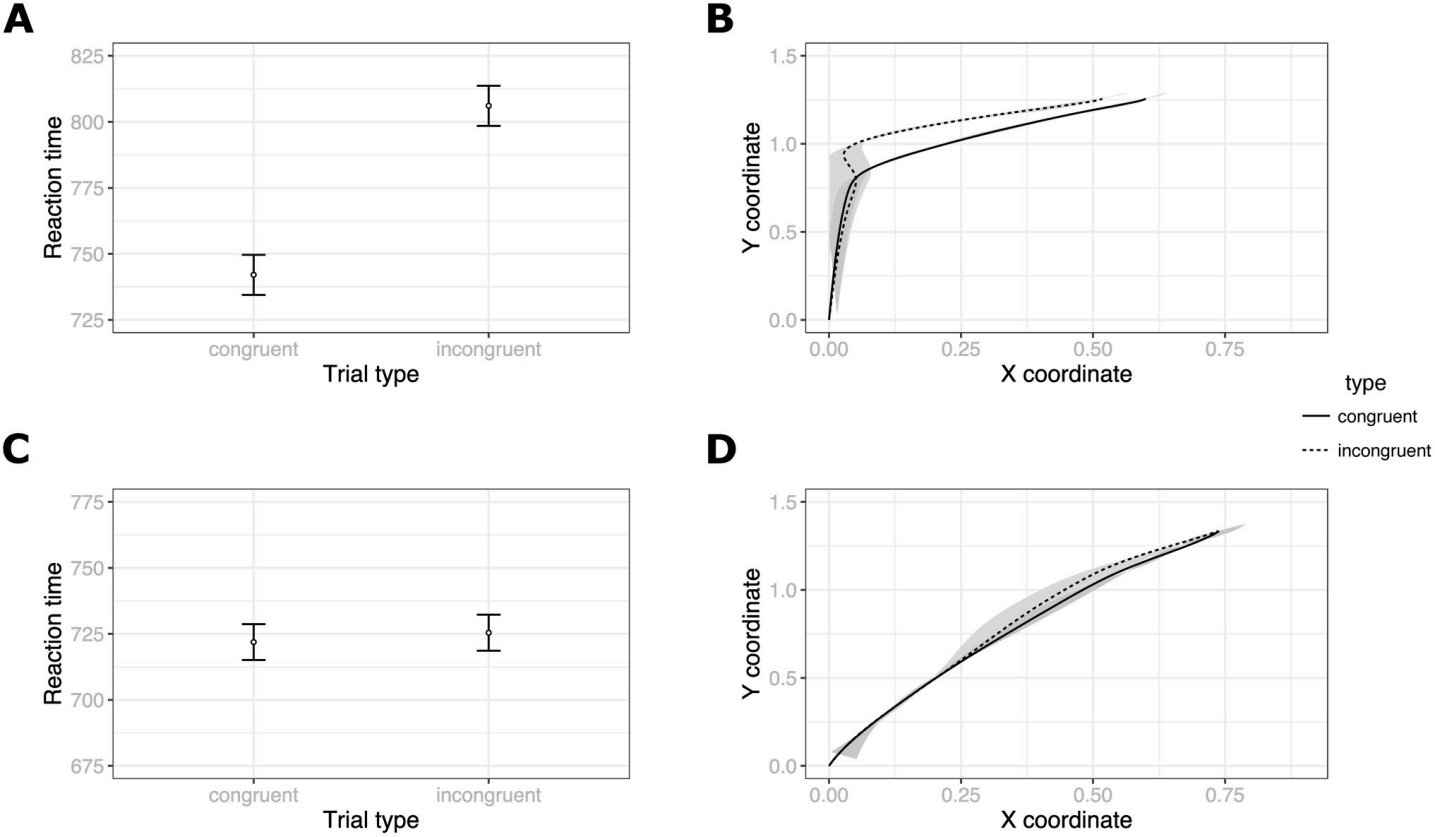
Results of Experiment 1 (A and B) and 2 (C and D). Reaction times with 95% confidence intervals are shown in figures A and C. Figures B and D show normalized movement trajectories averaged across participants with shaded areas corresponding to a 95% confidence interval on each coordinate.

Reaction time was measured as the time that elapsed between the moment participants clicked on the start button (indicating readiness to respond) and the moment they clicked on the response box. Participants took less time to complete Congruent (*M* = 742.07, *SD* = 73.62) than Incongruent (*M* = 806.09, *SD* = 65.39) trials (Fig 3A). A paired, one-tailed t-test showed that this difference was significant *t*(18) = −12.53, *p* < .001 and represented a large effect *r* = .95.

#### 2.2.2 Movement curves

The most widely used measure of trajectory attraction towards an unselected response is Area Under Curve, i.e., the size of the area between each trajectory and a straight line between the trajectory beginning and end point [38]. This measure can be obtained for all participant trajectories and then averaged across all trials to obtain a single value for each trial type in question. Fig 3B shows the shape of the average observed trajectories in Congruent and Incongruent trials. We again performed a paired one-tailed t-test on such aggregated AUC values and found that the curvature was significantly greater in Incongruent (*M* = .42, *SD* = .05) than in Congruent (*M* = .35, *SD* = .07) trials, *t*(18) = −6.8, *p* < .001, *r* = .85. Thus, this result mirrored the reaction time results. See S1 File. for inter-variable correlations for all experiments reported here—Tables 2 to 5 in S1 File.

### 2.3 Discussion

Our Experiment 1 demonstrated a standard individual mouse-tracking Simon effect as higher RTs and a greater movement curvature in Incongruent compared to Congruent trials. It therefore replicated part of the findings of the previous study by [34] for a different type of stimulus. Most importantly, the fact that we found the effect in both RTs and mouse trajectories and that it was large, gave us strong evidence that the particular mouse-tracking setup we implemented was successful. That is, the setup we used *can* lead to a Simon effect in the individual condition and therefore can be used to investigate whether an effect can be observed also in the joint condition in which the task is split between two participants (Experiment 3). Before proceeding to this test, however, we needed to investigate the individual Go-NoGo condition.

## 3 Experiment 2

Having found the Simon effect in reaction times and trajectory curvature in the individual Simon condition, we set out to find out whether we find an effect if participants are requested to respond only to one of the cues. According to previous studies, no effect of trial congruency should be found in Experiment 2.

An additional question concerned the behavior of participants in ‘passive’ trials, i.e. the trials in which the cue indicated that they were required to abstain from response rather than click on either of the response boxes. The advantage of mouse-tracking methodology here is that we can observe how a decision to inhibit the response guides the movement, which is not possible with a simple reaction time measure.

### 3.1 Methods

#### 3.1.1 Participants

Given the large effect sizes in Experiment 1, we kept the same number of participants. Thus, twenty participants from the Radboud University participants pool (18 women; ages between 20 and 51 years, mean age 26.5) were recruited for the second experiment. All were right-handed, had normal or corrected-to-normal vision and were not aware of the purpose of the experiment. None of the participants participated in the previous experiment. They were paid 10 euros for their participation. The study was approved by the institution’s local ethics committee (ECSW2015–1105-309) and written informed consent was obtained from each participant.

#### 3.1.2 Apparatus, stimuli, task and procedure

The apparatus, stimuli and procedure were as in Experiment 1. The task differed from the one employed in Experiment 1 as follows. Each participant was told to respond to only one of the color squares and refrain from responding to the other (“do nothing”). A click on a response box in such a trial was counted as an error. A trial ended upon a click or after the deadline (on successful NoGo trials). The remaining features of the task (time course of each trial, practice blocks, counter-balancing) were as in Experiment 1. Although participants were aware that the two gray squares in the upper corners of the screen were both response boxes and that a cue could be of two colors, they were not given any explicit indication of a complementary stimulus-response pairing.

#### 3.1.3 Data preprocessing

The data was pre-processed as in Experiment 1.

### 3.2 Results

As in Experiment 1, responses were coded as Congruent and Incongruent depending on the relationship between the location of the cue and the location of its assigned response box. However, given that participants were required to respond only to one of the cues, another variable was encoded that indicated whether a given trial was ‘active’ (required response) or ‘passive’ (required refraining from responding). Given that it is likely that different cognitive processes operated in active and passive trials, we have analyzed the corresponding data separately.

We removed a participant who gave a number of correct responses that was 3*SD* lower than the mean number of correct responses in the sample. Of the remaining data we removed trials as in Experiment 1 with an additional criterion. In a number of trials participants dipped the mouse cursor below the *y* = −0.1 coordinate in normalized space, which would cause issues with the package that was used for trajectory analysis. We therefore removed these trials (maximum 7 trials for a given participant). The filtering eliminated around 3.1% of the remaining data.

#### 3.2.1 Reaction times

Within active trial data, participants took comparable amount of time to complete Incongruent (*M* = 725.43, *SD* = 189.66) and Congruent trials (*M* = 721.88, *SD* = 178.91). According to a paired one-tailed t-test the difference was not significant *t*(18) = −0.77, *p* = .22, *r* = .18. Thus, as predicted from the literature, we found no effect of trial congruency on reaction times in the individual Go-NoGo condition (Fig 3C).

Since passive trials required no clicking response from the participant, they all took maximum amount of time (2s) and hence no explicit reaction time measure could be defined. We did, however, attempt to define an implicit reaction time measure as time that elapsed until the maximum y-coordinate was reached and started decreasing. The rationale behind this measure is that at the end of every trial participant had to return to the starting position in order to be ready for the next trial. When the cue required inhibiting the response, it would have been most efficient to simply return to the starting point. Greater conflict in either type of trials could cause a delay in response inhibition causing the turning point (the reversal from the highest reached y-coordinate) to occur later in the trial.

We found no effect of this type in passive trials, in which the time until the reversal from highest y-coordinate was similar for Congruent (*M* = 960.61, *SD* = 574.17) and Incongruent (*M* = 962.51, *SD* = 598.19) trials and the difference was not significant according to a two-tailed paired t-test *t*(18) = −0.12, *p* = .09. The effect could of course be masked by the huge variance in this data, which in turn could be explained by the large variety of motion trajectories exhibited by the participants, to which we turn next.

#### 3.2.2 Movement curves

**Fig 4:**
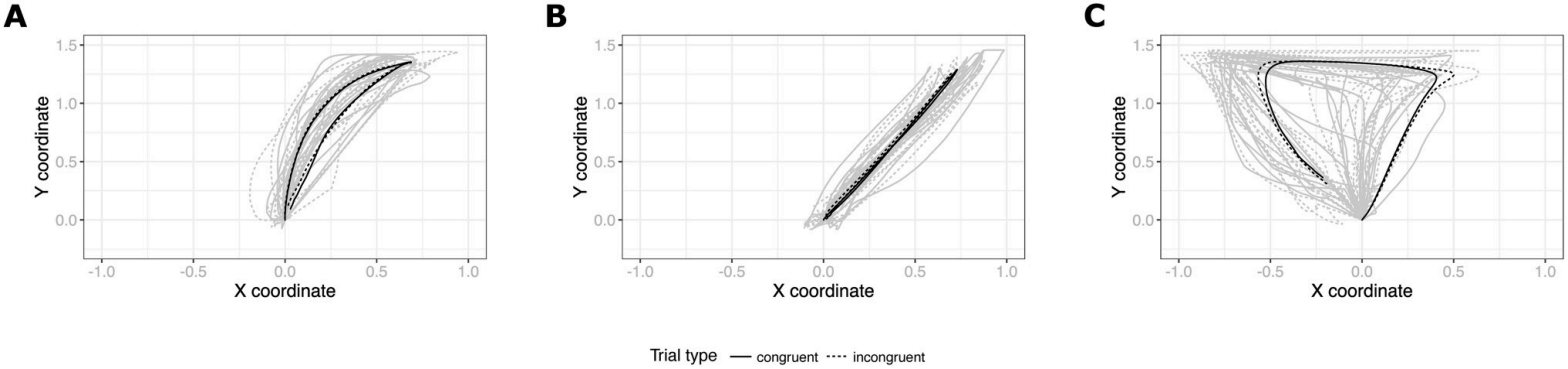
Example passive trajectories of 3 different participants in Experiment 2. Shown in black are average trajectories from the whole experiment duration. Shown in gray are particular trajectories from the last 40 trials. One can note that participants differ in how they go towards the response box—in a slightly curved manner (A) or in a straight line (B and C). They also differ in how they choose to return to the starting position—curved but staying in the same half of the screen (A), in a straight line (B) or making a circle through the opposite part of the screen (C).

The AUC calculated for participant trajectories in active trials was similar in Incongruent (*M* = .08, *SD* = .12) and Congruent (*M* = .07, *SD* = .1) trials. A paired one-tailed t-test showed that it was not significant, *t*(18) = −.96, *p* = .17, *r* = .22. Interestingly, movement trajectories differed qualitatively from the patterns observed in Experiment 1 (Fig 3D). While in Experiment 1 participants tended to move straight up first and only then turn towards the selected response, in Experiment 2 the movement would start directly towards the assigned response box (see also Figs 1–4 in S1 File for plots of individual average trajectories and Section 5 below for further discussion). Since the only difference in instructions in the two experiments regarded whether participants need to respond to both cues or only one, we can surmise that the movement strategy was freely adopted by the participants. Given that in Experiment 2 they had to click on only one of the response boxes (located on one side of the screen), they have chosen to always start moving towards that side.

We have also explored the movement patterns in passive trials. It became quickly apparent that trajectory curvature measures, such as AUC, would not be applicable because participants adopted very different strategies for dealing with the time they had when they were required to not click on the response box (see Fig 4). Given that passive data was not our primary concern and we found no effect in passive RTs, we decided not to investigate it further.

### 3.3 Discussion

Experiment 2 confirmed an absence of a Simon effect in an individual Go-NoGo version of the paradigm. We have also made a new observation, namely that people in this paradigm seem to adopt a qualitatively different movement strategy compared to the standard individual Simon task. That is, while in Experiment 1 participants moved straight up and then turned to a response box in a curved manner, in Experiment 2 participants tended to move to a response box in a straight line. This strategy intuitively makes sense, given that in the latter case they have only one response location throughout the whole experiment, while in Experiment 1 they have to continuously switch between two response locations. As we will show, this observation is relevant for interpreting the results of our last experiment.

## 4 Experiment 3

Experiment 1 confirmed the presence of a Simon effect when participants respond to both cues and Experiment 2 its absence when they perform only half of the task. We took this pattern of results to validate our experimental setup and proceeded to conduct Experiment 3 that employed a joint version of the task. In this version two participants share a task in such a way that each of them performs half of it, i.e. responds to one of the cues. According to the joint action literature, such a setup induces people to covertly co-represent the co-actor’s task and therefore we should expect a reappearance of a full-blown Simon effect. That is, in Experiment 3 participants should take longer to respond in incongruent trials and exhibit movement trajectories curved towards the incorrect response as in Experiment 1. Given the qualitative differences between movement patterns in the two previous experiments, we might add that should a full-blown mouse-tracking version of a JSE occur, we would expect trajectories to be more like in Experiment 1, i.e. directed upward and curved, rather than straight.

We were additionally interested in a possible modulating effect of perceiving the co-actor’s movements and therefore we implemented two conditions in Experiment 3, that we call Online and Offline condition. In the former, participants were able to observe the co-actor’s cursor moving on the screen in addition to their own throughout the experiment. In the latter, only one’s own cursor was visible. We made no specific predictions with respect to the difference between the Online and Offline conditions. On the one hand, given the relatively ‘offline’ nature of co-representation (should the effect occur), one could expect no difference between actually perceiving the co-actor’s movements versus merely knowing about their involvement in the task. On the other hand, perception of the co-actor might affect one’s own movements in two ways, either augmenting the feeling of ‘jointness’ of the action and therefore increasing the interference observable as a JSE, or instead making the division of labor more clear thereby decreasing the interference.

### 4.1 Methods

#### 4.1.1 Participants

Sixty participants from the Radboud University participants pool (48 women; ages between 18 and 54 years, mean age 24.4) were recruited for the third experiment. A sensitivity analysis conducted with G*Power [40] showed that this sample was sufficient to detect small effects, *f* ≥ .08 for our mixed effects ANOVA, assuming *α* of 0.05, power of 0.8 and correlation between repeated measures of 0.9 (see Tables 2–5 in S1 File for inter-correlation tables). All were right-handed, had normal or corrected-to-normal vision and were not aware of the purpose of the experiment. None of the participants participated in the previous experiments. They were paid 10 euros for their participation. The study was approved by the institution’s local ethics committee (ECSW2015–1105-309) and written informed consent was obtained from each participant. Participants were randomly assigned to the Online and Offline versions of the task. They were also randomly allocated into pairs, not constraining the couple composition by gender or age. None of the participants indicated previous familiarity with each other.

#### 4.1.2 Apparatus and stimuli

**Fig 5:**
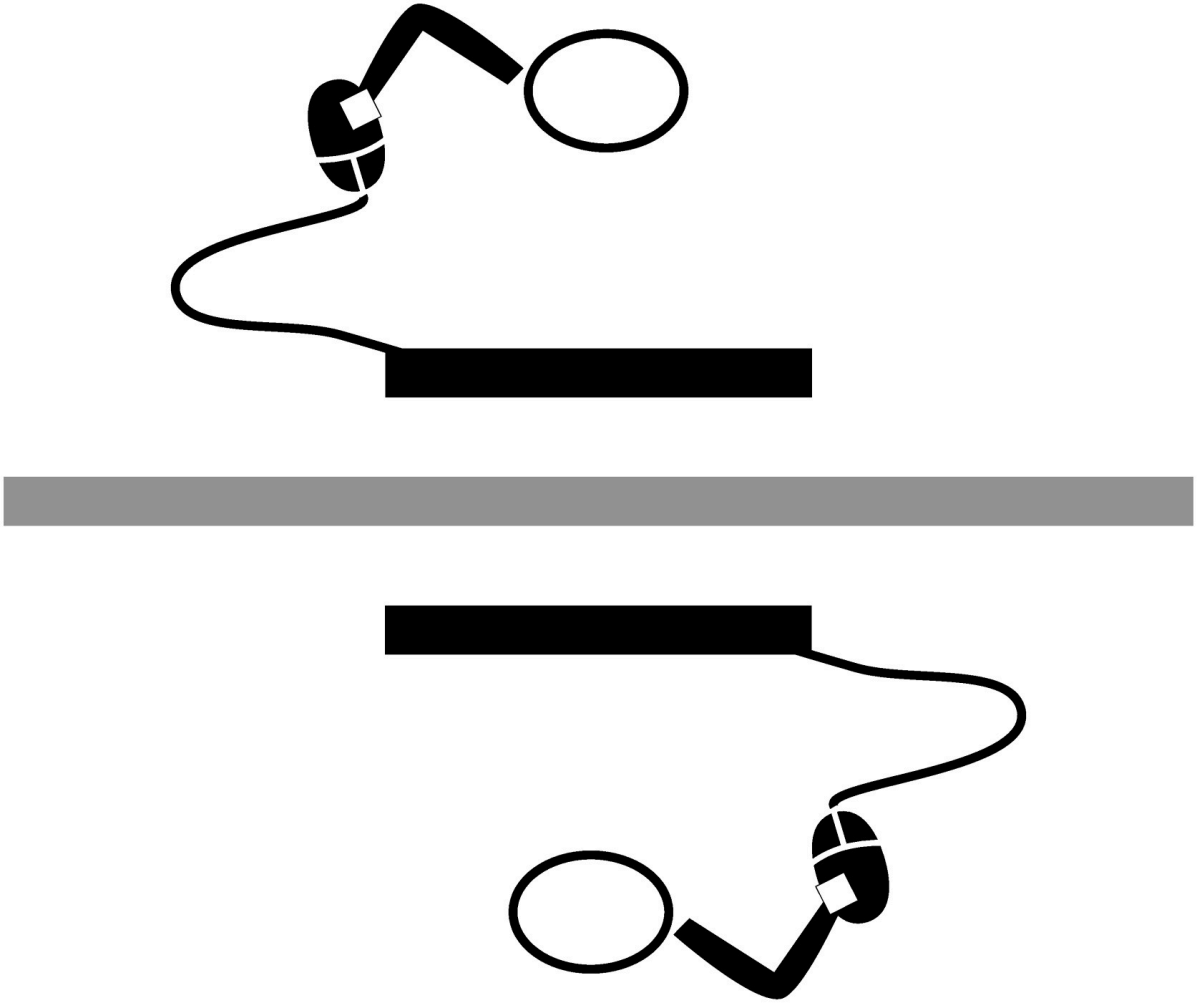
The physical setup of Experiment 3. Participants are sitting opposite each other separated by a partition wall.

The apparatus and stimuli were similar to those employed in Experiments 1 and 2. In both conditions participants were sitting in the same room facing towards each other (see Fig 5) with a partition placed between their desks. Each participant in the pair was seated in front of their own computer. The two computers were connected using a serial port that enabled two displays to be synchronized with each other in real time. The participants entered the room together, were aware of each other’s position and received instructions that made their respective cue-response pairings explicit.

#### 4.1.3 Task and procedure

The task and procedure were as in Experiment 1 with the following differences. The task was divided between the participants in each pair with one required to respond to blue and another to the red color of the cue. Regarding the time course of each trial, both participants had to click on the “start button” box and start moving upwards before the deadline in order for the trial not to be aborted. Since participants could respond at different speeds, the criterion for the deadlines was effectively based on the participant that responded last. Not responding or clicking on the incorrect response box was considered an error.

The only difference between the two joint conditions was the visibility of the mouse cursor of the co-actor (visible in the Online and not visible in the Offline condition). Own cursor was drawn in white while the cursor of the co-actor appeared in grey in order to facilitate discrimination between the two.

#### 4.1.4 Data preprocessing

The data was pre-processed as in Experiments 1 and 2. It must be noted that each trial required both participants to click on the start button within a deadline but they did not necessarily click at exactly the same time. We have chosen to align each participant’s trajectory to their own starting point and not to the starting point of the trial.

### 4.2 Results

As in Experiment 2, responses were coded for congruency and whether a participant’s role was ‘active’ or ‘passive’ in a given trial. Given the task division this means that in each trial the roles of the pair members were complementary.

Similarly to previous experiments, we removed participants with a large number of incorrect responses and trials with missed deadlines, errors, exceedingly long sampling intervals or total reaction time. As described in S1 File (pg. 1), we had to remove data from 2 participants in the Online and 3 participants in the Offline condition. However, while in the Online condition participants removed came from the same pair, in the Offline condition they came from different pairs. This happened because 3 individuals in 3 different pairs misunderstood the instruction and were responding to the location of the cue and not its color. Since most of the further analysis concerns active trials only, we removed complete data of these participants and passive trials of their co-actors (preserving the co-actors’ active trials). This resulted in the removal of 6.7% of the remaining data from the Online and 13.2% from the Offline condition.

#### 4.2.1 Reaction times

**Fig 6:**
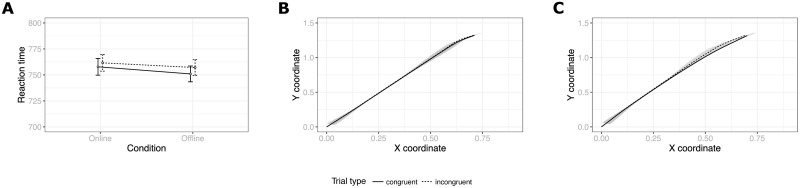
Results of Experiment 3. Reaction times (A) and average trajectories depending on trial type in Online (B) and Offline (C) conditions, shown with 95% confidence intervals.

We performed a 2 x 2 ANOVA with a between-subject variable Condition (Online vs. Offline) and a within-subject variable Trial type (Congruent vs. Incongruent). It showed that there were no significant differences between conditions or trial types, all *Fs*(1, 53) < 1.7, all *p* > .1, all $\eta_{p}^{2}<.05$. Summary statistics are presented in Table 1 (see also Fig 6A).

**Table 1:** Descriptive statistics in Experiment 3.

Condition	RT (ms)		AUC	
	Mean	SD	Mean	SD
Online-congruent	758	113	0.028	0.113
Online-incongruent	762	122	0.031	0.121
Offline-congruent	751	170	0.039	0.145
Offline-incongruent	757	178	0.05	0.16

#### 4.2.2 Movement curves

Paralleling the results of the RT data also the trajectory deviations measured as AUC did not exhibit significant differences between different conditions, all *F*(1, 53) < 2.7, all *p* > .1, all $\eta_{p}^{2}<.05$.

Visual inspection of trajectories averaged across participants indicates that they behaved as in Experiment 2, moving straight for their assigned target response box (Fig 6B and 6C) and not exhibiting any attraction toward the incorrect side.

#### 4.2.3 Velocity profiles

**Fig 7:**
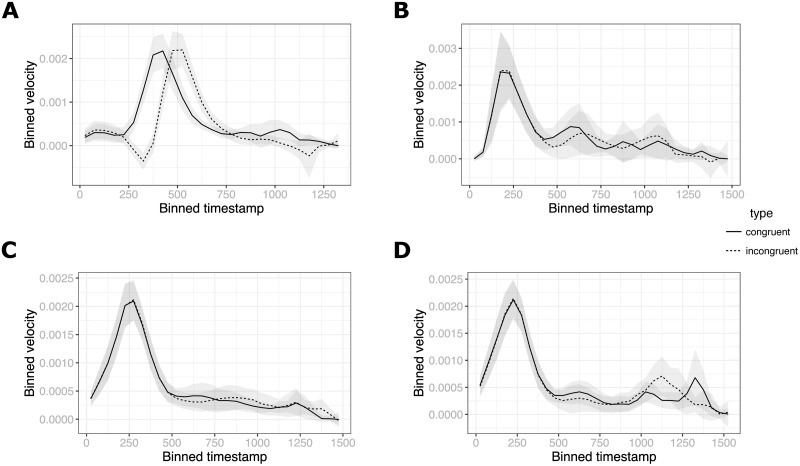
Binned velocity profiles in all experiments. Experiment 1 (A) shows a clear dissociation in time evolution of velocity between congruent and incongruent trials, especially in the beginning of the trial. Experiments 2 (B) and 3 (C: Online condition, D: Offline condition) show no difference.

The strength of the mouse-tracking methodology is that we can examine not just the topology of resulting movement trajectories but also how the movement unfolds in time. It has been suggested, for example, that “stronger competition between response options should be characterized by an initial decreased velocity as competing choices inhibit each other, followed by an increase in velocity once the system converges upon a decision and the inhibition is alleviated” [41]. This process would produce characteristic velocity profiles (i.e., movement velocity plotted over time) in Incongruent compared to Congruent trials and any notable differences could be subjected to further statistical analysis. Fig 7 shows plots we produced for all the experiments reported here by binning movement velocity into 50 ms bins and averaging over trials and over participants. It can be noted that while in Experiment 1 participants indeed seem to show a decrease in velocity in Incongruent trials, there is no such effect in Experiments 2 or 3. While this simple visualization does not exhaust more sophisticated approaches to analyzing response dynamics that could be applied to our data, the result is in line with the measures presented above.

### 4.4 Discussion

In Experiment 3 we found no mouse-tracking JSE. This absence of a JSE held regardless of the visibility of the co-actor’s movements, and is contrary to the prediction based on the presumed pervasiveness of co-representation. Also, given the strong Simon Effect observed in trajectories in Experiment 1 we were surprised to find no effect in Experiment 3, not even in the online condition. Notably, the overall shapes of the average trajectories were quite different from those observed in Experiment 1 and more similar to those in Experiment 2 (i.e., ‘straight’ trajectories moving in a straight line directly to the response box rather than ‘curved’ trajectories that moved up first before curving towards the response box).

A closer inspection of individual trajectories did reveal, however, that participants moved their mouse in qualitatively different ways, some in fact displaying ‘curved’ rather than ‘straight’ trajectories, suggesting that they may have been using different strategies for performing the task. To probe to what extent the individuals that displayed curved trajectories may show a JSE at the individual level (which may have been masked in the group-level analysis by the majority of ‘straight’ trajectories), we decided to perform individual analyses to better characterize individual strategies and test for JSE at the level of individuals. The results of this additional analysis are reported next.

## 5 Individual-level analysis

**Fig 8:**
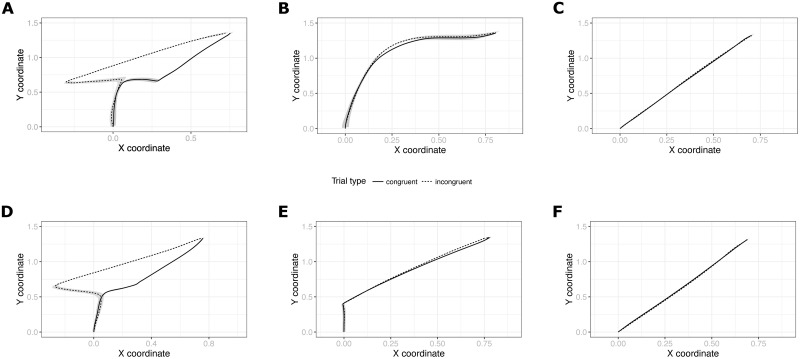
Example average individual movement trajectories in active trials in Experiment 3 (A-C) and 2 (D-F). Plots A and D show curved participants with a detectable Simon effect. Plots B and E show curved participants without a detectable Simon effect. Plots C and F show straight participants without a detectable Simon effect.

In order to survey the potential individual differences in movement patterns exhibited by the participants of our Experiment 3, we plotted trajectories averaged per participant instead of averaging across them. We noticed that in fact several participants (3 persons in Online and 4 in Offline) seemed to adopt a movement pattern more similar to those observed in Experiment 1, i.e. moving first straight up and then curving towards the response box. The examples of ‘curved’ and ‘straight’ average trajectories of Experiment 3 are shown in Fig 8A–8C. Plots of all individual trajectories from Experiments 1–3 are available as Figs 1–4 in S1 File.

**Fig 9:**
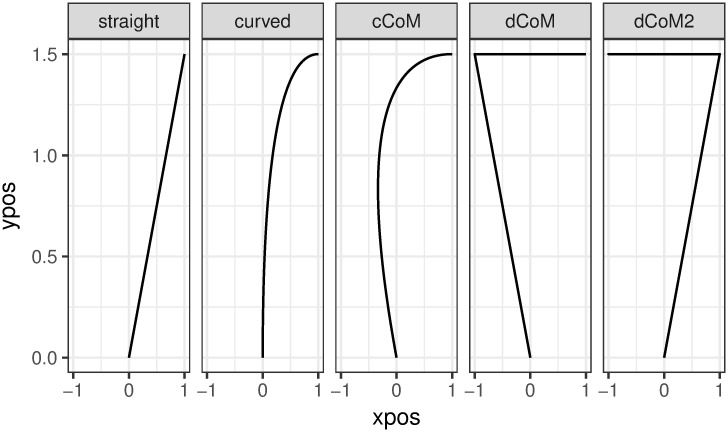
Trajectory prototypes. Straight, mildly curved, continuous change-of-mind, discrete change-of-mind, and double discrete change-of-mind.

We proceeded to investigate whether people with a majority of curved trajectories show a JSE on the individual level. To that end, we first applied a clustering technique to automatically determine which participants exhibited mostly curved and which mostly straight trajectories. We have used a function provided by an R mousetrap package [39] that calculates geometric distance (Euclidean distance by default) between each trajectory and a number of predefined prototype trajectories (depicted in Fig 9) and then assigns a label that represents the closest prototype to that trajectory. We then collapsed three of the default prototypes (‘curved’, ‘cCoM’ and ‘dCoM’) that have a curved shape to a single ‘curved’ category, removed trials that were assigned a very infrequent prototype (‘dCoM2’ occurred in 0.1% of the cases) and calculated for each participant how many straight and curved trajectories they produced. Finally, each participant was assigned to a Straight or Curved category based on a simple majority of their trajectory types. The automatic classification confirmed our intuitive observations.

After classifying our participants, we went on to run a series of independent one-tailed t-tests on the trial data from all the participants to see whether an effect could be detected at the individual level. We were specifically interested if a possible effect was differentially present for curved and absent for straight participants. We adopted a significance level of 0.05/2 = 0.025 as our cut-off, correcting for a double comparison conducted on each individual (one for the group test, one for the individual level).

The tests revealed that 3 curved-strategy participants in Experiment 3 exhibited a significant JSE in their RTs and one of them additionally in their trajectory curvature. From the 48 straight-strategy participants only one showed a significant difference in AUC measure depending on the trial type. However, the effect was based on a negative area under curve meaning that the deviation of that participant’s average trajectories in incongruent trials was *away* from the alternative response.

Having found an individual-level significant JSE in Experiment 3, we felt it appropriate to repeat this procedure also for the data from Experiment 2, where we had concluded an absence of an effect based on a group-level analysis. We therefore plotted individual average trajectories of participants from Experiment 2 (see examples in Fig 8D–8F), mapped them to curved vs straight prototypes and run a series of t-tests. The procedure established that indeed 5 of 19 participants were assigned to the Curved category and 2 out of these 5 exhibited a Simon effect in their RTs and AUC measure. None of the straight-strategy participants showed such an effect.

## 6 General discussion

In our series of three experiments we have found a strong Simon Effect in both reaction times and mouse trajectory curvatures in the Standard individual Simon Task (Experiment 1), no effect in the individual Go-NoGo task (Experiment 2) and no effect in the Joint Simon Task—the latter regardless of the visibility of the co-actor’s movements (Experiment 3). Additionally, we found evidence for individual variation in movement strategies adopted by participants in conditions in which response to only one of the two stimuli was required (Experiment 2 and 3).

The pattern of results in all three experiments suggests that the Joint Simon Effect does not seem to readily generalize to a more continuous response modality, and hence co-representation may not be as pervasive and automatic as previously thought. As always, however, a null result needs to be treated with care and therefore we consider its possible interpretations as well as several limitations of our study.

First of all, one could object that our lack of the JSE could be due to a failure of the experimental paradigm. For example, some studies have found the effect only when participants sat next to each other, rather than away from each other [25], or only under conditions of a compatible spatial reference frame of the co-actor [42]. One may argue that this could potentially provide an explanation for the lack of the JSE from a domain-general perspective since what matters there is that the co-actor provides a salient spatially defined distractor but in our study the co-actor’s body is not salient in the left-right spatial dimension (since they are sitting in front of the participant) and the co-actor’s response is actually to the same side of the room from the bird’s eye view. However, in our joint condition (especially in its Online version) there was a salient event of the co-actor’s cursor moving towards the response box on the side of the screen opposite to the participant’s own response box. In the absence of a way to determine a priori which events are crucial to elicit a JSE from the domain-general perspective—the co-actor’s physical presence, their imagined physical actions or their action effects on the screen—it remains unclear why a screen-based salience is insufficient. Especially given the recent finding that merely an experimenter’s presence in the room might produce a Simon effect type of modulation [43] Furthermore, while these factors could be cited by the proponents of the domain-general account, we think there is no basis in the social account for rejecting our experimental setup. Co-representation is supposed to arise due to the knowledge of the task. The participants in our study were explicitly informed that they are doing the task together and their respective parts of the task were made clear. Thus, we see no reason from the social perspective to expect a lack of the effect in the setting we employed, Unless the proponents of the social account were to postulate that co-representation is based on simulation, in which case compatibility of a reference frame would be a relevant factor. We know of no explicit argument to this effect. In fact, the lack of effect is especially puzzling given that the JSE has been found in the past with participants sitting in separate rooms [4, 18]. That being said, future studies could look at the influence of physical factors on the mouse-tracking JSE.

We further believe that other elements independently support the validity of our paradigm. Most importantly, we do find an effect in the individual Simon task (Experiment 1). If co-representation is a process that mirrors executing the task on one’s own, it should also emerge in exactly the same setting in which it is split across participants. In addition, it appears that several people in Experiment 3 (specifically those who adopt curved movement strategy) do show an effect at the individual level, suggesting that the paradigm is sensitive enough to detect a JSE if it occurs. For example, if most of the participants had adopted the strategy that generates curved trajectories (presumably based on co-representation, on the social account), we would arguably have found a full-blown JSE at the group-level. One could now argue that perhaps participants needed to be forced to move upwards (for example by placing a target in the middle of the screen that they need to pass through before moving to their response box). However, we see no theoretical reason why co-representation should hinge on forcing a particular movement strategy on the participants, as the theoretical link typically goes the other way—from knowledge of the task constraints to co-representation to particular response patterns.

With the above considerations out of the way, we need to emphasize that even if our conclusion of non-pervasiveness of co-representation holds, it does not imply that the phenomenon of co-representation does not exist in general. It might occur in a variety of settings, and in fact on the basis of our findings we cannot argue against inferences towards co-representation based on JSEs obtained in the past in other paradigms. One should note that the features of co-representation that we discussed in the Introduction (its social and offline nature, as well as pervasiveness) are logically not a package deal. JSEs could be a result of a co-representation-based mechanism and such co-representation could be a real phenomenon that plays an important role in joint action without it being automatic or pervasive. The tendency in the social account has been to argue that (1) it is an ‘offline’ mechanism, inducible by mere knowledge of the task and its division and (2) that it is automatic, activated even in contexts in which it is not beneficial to the overall behavior. From these two points, it has been assumed that the pervasiveness claim follows: co-representation occurs in many situations, for example, including those that are more ‘online’, in which not only is there a knowledge of the task but also perceptual information about the co-actor. One could claim, however, that co-representation is social and important but restricted to certain type of situations.

Viewed in this light, our study contributes to testing the boundaries of co-representation. Had we found that JSE occurs in Experiment’s 3 Offline but not Online condition, we could have concluded that perhaps co-representation is restricted to cases in which there is knowledge of the joint task but no perceptual information about the co-actor’s movements. Since we found no JSE in either of the Experiment 3 versions, we need to conclude that it is rather something about the continuous response modality that weakens the tendency to co-represent. A tentative explanation for this that we would like to propose is something like ‘offloading’ of cognitive processes onto the resources of the body and the environment [44]. That is, given that participants in Experiment 2 and 3 only need to respond to one side of the screen throughout the experiment, they are able to stabilize the movement towards that side by adopting a straight strategy from the very beginning of each trial. Once the strategy is settled, the option to move to the opposite side is removed and therefore the incongruent location of the cue no longer influences the execution of the responses. At this point this explanation is speculative but we think it warrants further probing in future studies.

We think the most interesting result of this study is a discovery of qualitative individual differences in how people approach the task. First, we think it points to an interesting methodological point. Namely, the individual differences are readily apparent from a plot of individual average trajectories while at the same time not being always discernible from the RT data of the same participants. This raises the possibility that also in past studies there is less uniformity than previously assumed that remains hidden by using a coarser response modality. We hope future research will complement traditional methods with designs that can detect individual differences and incorporate considerations of variation into discussion of potential mechanisms behind the effects. Mouse-tracking methodology in particular has a big advantage for addressing this type of questions.

Second, the fact that we also find curved trajectories and individual-level effects in Experiment 2 poses an interpretative difficulty. It seems that even in the absence of the social context some people are affected by the complementary task rule. The reason for this is unknown in the present study. It could be that those participants spontaneously decide to imagine responding to the alternative cue (which has been shown to produce the Simon effect in the individual Go-NoGo condition; [27]). It could be that some participants’ attention is drawn toward the stimulus location influencing the resulting movement despite it being irrelevant (see Fig 8D). Regardless of the precise mechanisms responsible for these effects in Experiment 2, it certainly suggests that similar effects in Experiment 3 (i.e. individual-level JSE and curved movement patterns) could be driven by the same processes and therefore not be ‘social’ in nature. Alternatively, different mechanisms could be responsible for similar looking patterns in Go-NoGo and Joint versions of the paradigm. Perhaps despite superficial similarity there are other differences between them not captured by the RT and AUC measures (e.g. differences in trajectory angles, movement entropy or behavior on passive trials). Future studies should more rigorously compare these conditions.

Finally, if it were to be established in the future that the effects in Experiment 3 (namely, the lack of a JSE) are genuinely social, we think it could raise another interesting possibility: that joint action can be accomplished using a variety of strategies. The participants could have adopted their straight trajectories because it facilitated a division of labor coordination by dividing the space assigned to each member of the pair. That is, one could still be in the presence of a *joint* action rather than participants merely performing individual halves of the task in parallel, even if no JSE is found. This of course would lead to the question what differentiates between no JSE because participants are simply ignoring the co-actor and no JSE because they are adopting a division of labor strategy (and therefore are still in a way acting jointly). Perhaps also here other types of measures could capture the difference between these two situations, such as those that look at coupling between participants [45]. That is, behavior in a social situation could produce the same type of movement trajectories as behavior in an individual task (as captured by trajectory deviation) but, for instance, a correlation between participant trajectories within a pair could be higher than between a member of one pair and a member of another pair. This would mean that individual movement is affected on a very subtle level by the co-actor even if it does not show up as a curvature difference between congruent and incongruent trials.

To conclude, our results cast doubt on arguments for pervasive and automatic co-representation in joint action. Instead, they raise a number of questions for further research and prompt further refinement of joint action theories. On the one hand, they suggest that co-representation could be restricted to certain kinds of settings, like those that do not involve continuous movements, limiting its presumed pervasiveness and relevance for everyday action coordination. On the other hand, they raise the possibility that joint action can be accomplished by different strategies such as division of labor when afforded by the task conditions.

## Supporting information

S1 File Additional tables and figures. The file contains a table listing data exclusion criteria and corresponding numbers of removed participants and trials, inter-variable correlation tables and plots of individual trajectories in all 3 experiments.
(ZIP)
