# Supplementary material for: Joint Simon effect in movement trajectories
Source: PLoS One. 2021 Dec 29;16(12):e0261735. doi: 10.1371/journal.pone.0261735 (PMC8716062; doi:10.1371/journal.pone.0261735)
Supplement: S1 File — The file contains a table listing data exclusion criteria and corresponding numbers of removed participants and trials, inter-variable correlation tables and plots of individual trajectories in all 3 experiments. (ZIP) [file pone.0261735.s001.zip › S1_Supporting_Information.pdf]

Table 1: Data removed in different conditions

Condition	#Part	#Part rem	#Trials	Bad resp	Bad part	Bad intvl	Bad y-crdt	Slow trials	#Trials rem	%Trials rem
Exp1	20	1	12160	361	0	1	0	247	609	5
Exp2	20	1	12160	167	0	4	44	163	378	3.1
Exp3:Online	30	2	17920	732	0	301	22	153	1208	6.7
Exp3:Offline	30	3	17280	1240	460	368	33	184	2285	13.2

#Part: Number of participants in the experimental condition; **#Part rem**: Number of participants whose data was removed completely (see explanation below); **#Trials**: Number of trials remaining after removing participants; **Bad resp**: Trials removed because wrong response or no response was given; **Bad part**: Trials removed complementary to trials removed in the previous step (see below); **Bad intvl**: Trials with bad inter-sample intervals; **Bad y-crdt**: Trials in which y-coordinate dropped below a start button; **Slow trials**: Trials with duration longer than 3 median absolute deviations above the median; **#Trials rem**: Trials removed in total; **%Trials rem**: Percentage of trials removed (# Trials removed) out of trials remaining after removing complete participant data (# Trials).

Participants whose data was completely removed from Experiment 1, Experiment 2 and Experiment 3 Online condition had a markedly larger number of unsuccessful trials than other participants indicating they must have misunderstood the instructions or were unable to respond in time. Participants whose data was completely removed from Experiment 3 Offline condition gave correct responses in most of congruent trials but none in the incongruent trials. Investigating the trajectories of these participants further revealed that they were responding to the location of the cue and not its color. We removed complete data of these participants and also complementary passive trials of their co-actors.

The remaining columns show the number of trials removed for different reasons from the set remaining after deleting complete participants data. The high number of trials removed in Experiment 3 Offline condition is explained by the removal of passive trials described above and recording issues.

Table 2: Inter-variable correlations in Exp 1

	RT congr	RT incongr	AUC congr	AUC incongr
RT congr	–			
RT incongr	.96***	–		
AUC congr	-.55*	-.6**	–	
AUC incongr	-.48*	-.35	.72***	–

Table 3: Inter-variable correlations in Exp 2

	RT congr	RT incongr	AUC congr	AUC incongr
RT congr	–			
RT incongr	.996***	–		
AUC congr	.45	.46*	–	
AUC incongr	.7***	.71***	.91***	–

Table 4: Inter-variable correlations in Exp 3, Online condition

	RT congr	RT incongr	AUC congr	AUC incongr
RT congr	–			
RT incongr	.97***	–		
AUC congr	.4*	.54**	–	
AUC incongr	.4*	.54**	.997***	–

Table 5: Inter-variable correlations in Exp 3, Offline condition

	RT congr	RT incongr	AUC congr	AUC incongr
RT congr	–			
RT incongr	.99***	–		
AUC congr	.36	.38	–	
AUC incongr	.49**	.52**	.96***	–

RT = reaction time, AUC = area under curve,
congr = congruent, incongr = incongruent
*$p < 0.05$, **$p < 0.01$, *$p < 0.001$

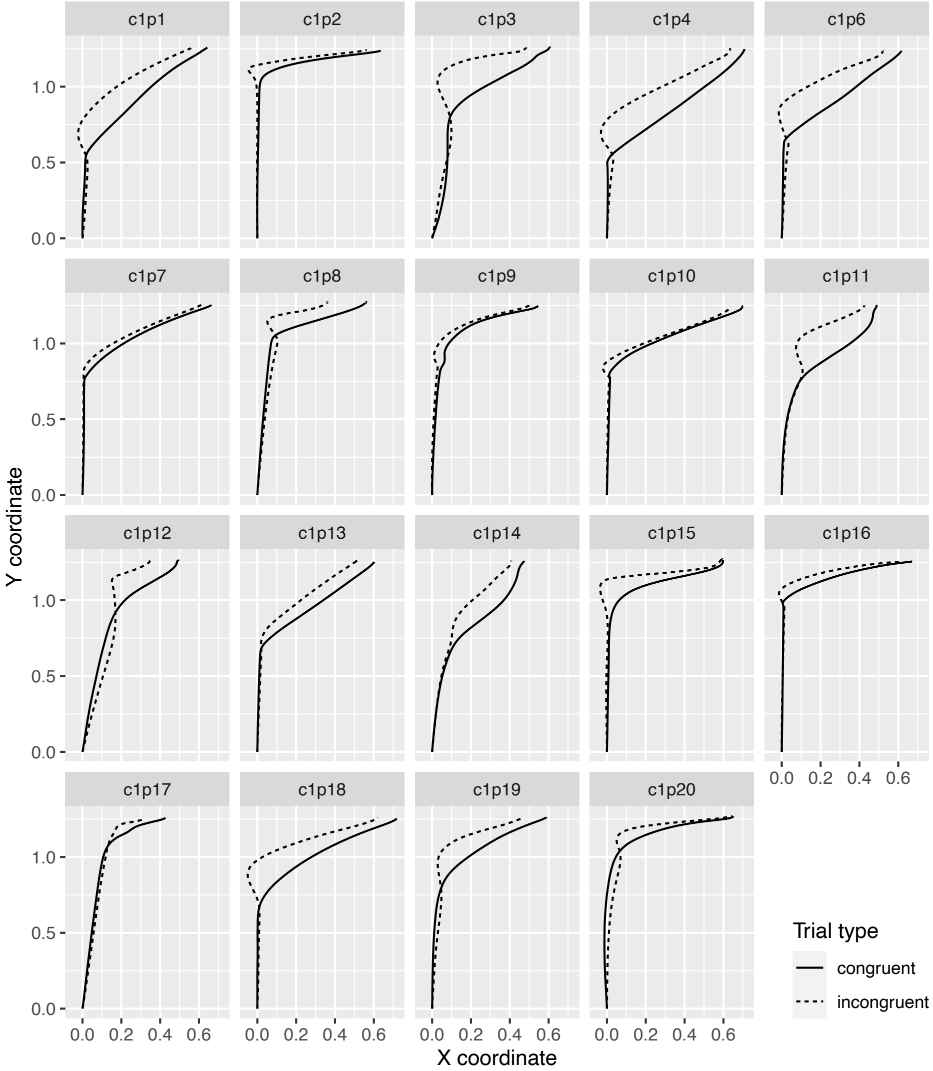

Figure 1: Individual average trajectories in Experiment 1.

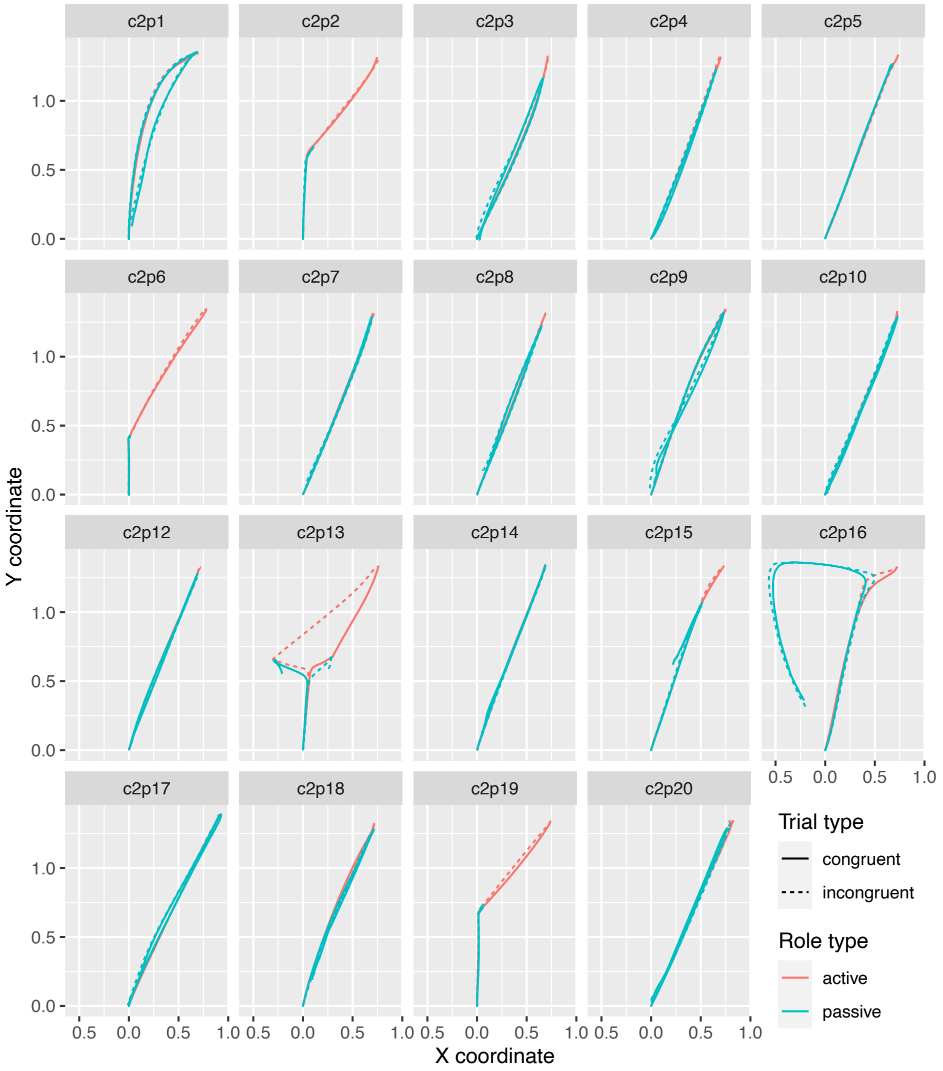

Figure 2: Individual average trajectories in Experiment 2.

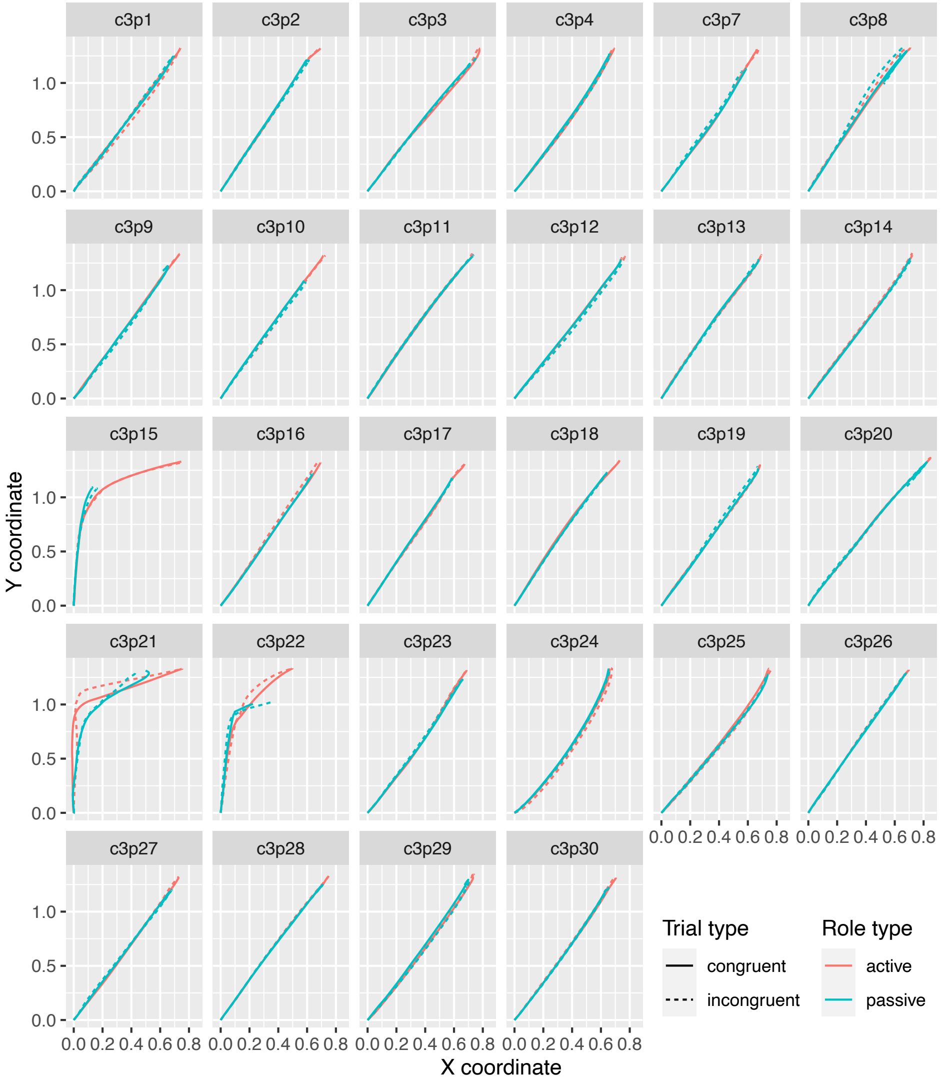

Figure 3: Individual average trajectories in Experiment 3: Online condition.

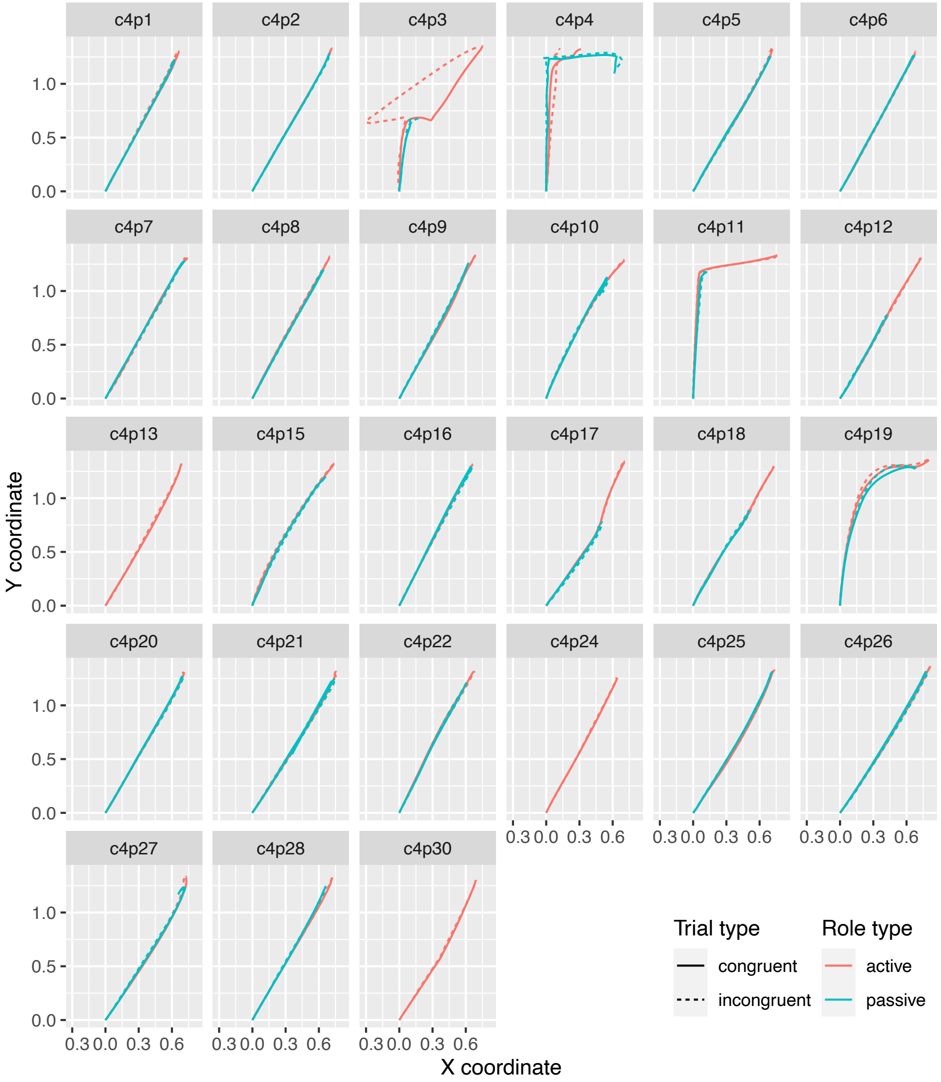

Figure 4: Individual average trajectories in Experiment 3: Offline condition.
